# Aperiodic Subthalamic Dynamics Distinguish Rest from Affective Processing: Implications for Adaptive DBS

**DOI:** 10.1192/j.eurpsy.2026.10714

**Published:** 2026-07-31

**Authors:** S. Karim, V. Voon, S. Sonkusare

**Affiliations:** Department of Psychiatry, University of Cambridge, Cambridge, United Kingdom

## Abstract

**Introduction:**

The subthalamic nucleus (STN) is central to basal ganglia circuits, integrating motor, cognitive, and limbic functions. Its aberrant activity underlies core motor symptoms of Parkinson’s disease (PD). While its motor role is well established, evidence implicates the STN in affective processing. Deep brain stimulation (DBS) alleviates PD motor symptoms and, perioperatively, allows intracranial recordings from implanted electrodes. These recordings provide a unique opportunity to study STN dynamics across brain states. Neural signals contain oscillatory components and a non-oscillatory aperiodic component in the background. This aperiodic component follows a **1/f** distribution, with two features: the **exponent**, the slope reflecting the level of local **excitation–inhibition balance**, and the **offset**, the overall broadband power (see Img. 1). Despite detailed STN oscillation studies, the aperiodic component remains under-investigated during emotion processing.

**Objectives:**

We aimed to characterise STN aperiodic features—the exponent and offset—during rest and **emotional stimulus** engagement, and assess their potential as markers of state-dependent excitation–inhibition balance.

**Methods:**

We analysed bilateral STN recordings from **19** PD patients (6 female; mean ± SD age = 59.7 ± 11.8 years) undergoing DBS. Recordings were obtained during rest and presentation of emotionally salient images from the International Affective Picture System (IAPS). Data were visually inspected and cleaned, then processed using the FOOOF algorithm to isolate the aperiodic exponent and offset. This yielded **114** electrode-level recordings across patients, which were analysed at the group level using paired t-tests (α = 0.05) between resting and task conditions.

**Results:**

The aperiodic exponent was lower during emotional processing **(1.44 ± 0.04)** than at rest **(1.49 ± 0.04**; t(113) = 1.68, **p = 0.03**). The offset was also lower during the task **(0.159 ± 0.059)** than at rest **(0.211 ± 0.053)**, but this was not significant (t(113) = 2.18, **p = 0.09**).

**Image 1:**
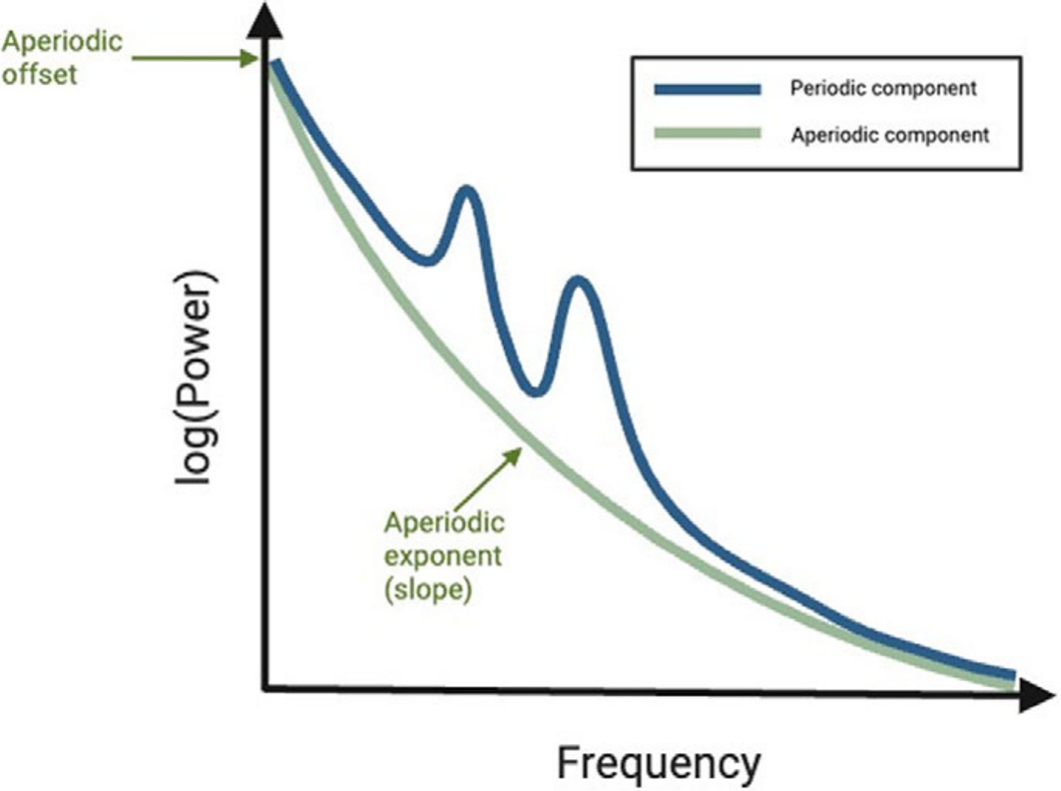

**Conclusions:**

We provide novel evidence that STN aperiodic dynamics are state-dependent and sensitive to emotional engagement. The decrease in the aperiodic exponent, corresponding to a **reduced 1/f slope**, reflects **increased excitatory activity** relative to inhibition, likely driven via hyperdirect cortical or indirect striatal projections. Further studies should examine differences between positive and negative emotional valence and associations with mood symptoms in PD. Though preliminary, these findings suggest the aperiodic exponent could serve as a biomarker of STN excitation–inhibition balance for adaptive DBS and a quantifiable metric to track disease progression and treatment response.

**Disclosure of Interest:**

None Declared

